# Penumbra: A spatially distributed, mechanistic model for simulating ground-level incident solar energy across heterogeneous landscapes

**DOI:** 10.1371/journal.pone.0206439

**Published:** 2018-12-19

**Authors:** Jonathan J. Halama, Robert E. Kennedy, James J. Graham, Robert B. McKane, Brad L. Barnhart, Kevin S. Djang, Paul B. Pettus, Allen F. Brookes, Patrick C. Wingo

**Affiliations:** 1 Environmental Science, Oregon State University, Corvallis, Oregon, United States of America; 2 U.S. Environmental Protection Agency, Corvallis, Oregon, United States of America; 3 College of Earth, Ocean, and Atmospheric Sciences, Oregon State University, Corvallis, Oregon, United States of America; 4 Department of Environmental Science and Management, Humboldt State University, Arcata, California, United States of America; 5 CSRA, Corvallis, Oregon, United States of America; University of British Columbia, CANADA

## Abstract

Landscape solar energy is a significant environmental driver, yet it remains complicated to model well. Several solar radiation models simplify the complexity of light by estimating it at discrete point locations or by averaging values over larger areas. These modeling approaches may be useful in certain cases, but they are unable to provide spatially distributed and temporally dynamic representations of solar energy across entire landscapes. We created a landscape-scale ground-level shade and solar energy model called Penumbra to address this deficiency. Penumbra simulates spatially distributed ground-level shade and incident solar energy at user-defined timescales by modeling local and distant topographic shading and vegetative shading. Spatially resolved inputs of a digital elevation model, a normalized digital surface model, and landscape object transmittance are used to estimate spatial variations in solar energy at user-defined temporal timesteps. The research goals for Penumbra included: 1) simulations of spatiotemporal variations of shade and solar energy caused by both objects and topographic features, 2) minimal user burden and parameterization, 3) flexible user defined temporal parameters, and 4) flexible external model coupling. We test Penumbra’s predictive skill by comparing the model’s predictions with monitored open and forested sites, and achieve calibrated mean errors ranging from -17.3 to 148.1 μmoles/m^2^/s. Penumbra is a dynamic model that can produce spatial and temporal representations of shade percentage and ground-level solar energy. Outputs from Penumbra can be used with other ecological models to better understand the health and resilience of aquatic, near stream terrestrial, and upland ecosystems.

## Introduction

Solar energy is generally the largest source of energy present in ecosystems and therefore must be well characterized in ecological models of those systems [1]. However, ecological models rarely include representations of spatially and temporally distributed solar energy. It is well known that solar energy that reaches the top of Earth’s atmosphere varies predictably with both location and time of day. Landscape objects may intercept portions of this energy as it travels towards the surface of the earth. Shade, or the removal of some portion of incident solar energy, that is projected on the earth’s surface has high spatial and temporal variability. Important components that generate shade at a given location include both proximal and distal topography, surface structures such as buildings and vegetative canopies.

When attempting to accurately estimate ground-level incident solar energy, a core challenge is retaining sufficient detail to accurately model the system in a computational model that is tractable to parameterize and run. Retaining detail is necessary because spatiotemporally inaccurate representations of solar energy or shading may cause compounding effects and introduce unknown modeling uncertainty in ecological models. For example, site specific climate station data is often used to homogeneously represent large spatial extents, though small-scale forest or stream system microclimates may greatly vary over short distances [2]. Accurate representations of environmental drivers are especially crucial at scales for which processes are sensitive to microclimate variability [3]. Models that produce dynamic spatially distributed solar energy representation require a balance between computational runtime, light complexity, and the volume of model output [4]. Stakeholders focusing on landscape restoration and management need to simulate ground-level solar energy across a wide range of both spatial and temporal scales in a computationally tractable fashion.

In this paper we present Penumbra, a landscape-scale ground-level shade and incident solar energy model designed to balance complexity with tractability. Penumbra results are presented using the 3D visualization tool VISualizing Terrestrial-Aquatic Systems (VISTAS) [5]. To test Penumbra’s predictive skill, we compare Penumbra results against open versus forested sites from the Environmental Protection Agency (EPA) Oregon Crest-to-Coast Environmental Monitoring transect (O’CCMoN) dataset [6]. Finally, we provide directions for future research by discussing Penumbra’s intended use, and the potential for integration with other spatiotemporal ecological models.

### Current approaches

Here, as a point of contrast to our modeling approach, we broadly address the existing methods that most ecological models use to represent solar energy. Simulation methods used to account for solar energy in ecological models range from simple aspatial approaches through spatially implicit to spatially explicit ray tracing. The simplest approach utilizes solar energy for a single location, then allocates that value to the entire landscape. This approach models clear-sky solar energy or uses observed sensor data without any spatially distributed reductions caused by topographic or landscape object shadowing. The spatially implicit approaches uses the same basic extrapolation strategy, yet constrains the areas for which the extrapolation occurs into groups that collectively represent the entire watershed or landscape of interest. An example would be a moderate size watershed (> 100 km^2^) divided into a series of stream segments or sub-watershed catchments. The data would represent spatially heterogeneous solar energy at the watershed full extent, yet each stream reach or sub-catchment would receive a uniform value. If each catchment actually consisted of unvarying tree coverage and landscape slope characteristics, that uniform value could be representative of the catchments true ground-level solar energy. Though computationally tractable, these approaches ignore the landscape variability present in most ecological systems.

Ray tracing models take a complex, spatially explicit approach. They simulate light rays’ and their object and landscape interactions, that is, tracking discrete light path reflection, refraction, transmission, and absorption for each simulation timestep [7–8]. This method closely mimics light, but at high computational expense due to the number of light rays needed to model a landscape and the potentially large number of landscape object interactions per ray. Even over a small spatial extent at a small spatial grain, the computational requirements per simulation can become burdensome [9].

Ideally, spatial models simulating shade or solar energy for stream networks or entire landscapes must balance promptness, temporal capability, and spatial extent. The simplest methods tend to misrepresent the spatial complexity of solar energy, while complex methods introduce excessive runtime and memory costs over moderate to large landscapes. Therefore, an ecological light simulation model is needed that can address the limitations of current light simulation methods. With moderate landscapes in mind (> 100 km^2^), an ideal landscape-scale solar energy model would address the following needs:

- Produce comprehensive ground-level incident solar energy and shade fraction estimates as a function of spatiotemporal variations in incoming radiation and shadowing by both topographic and landscape objects.
- Minimize user burden through relatively limited model inputs and parameterization.
- Provide flexible temporal runtime parameters to simulate landscape solar energy at user-required temporal and spatial resolutions (minutes to days; plots to regional landscapes).
- Develop flexible model coupling (loose and tight) for external model integration.

The resulting model should be able to assess the major components that affect incident ground-level solar energy across spatially heterogeneous landscapes in a spatially distributed manner. The model must perform within reasonable simulation runtimes to avoid user burden or severely extending runtime requirements when coupled with another ecological model.

### Research goal

To address these needs, we developed Penumbra, a new ground-level shade fraction and incident solar energy model. Penumbra is a direct solar energy reduction model, meaning the quantity of incoming solar energy is reduced due to the spatial structure of the environment being simulated. The resulting shade and solar energy data values are the remaining fraction of shade and incident ground-level solar energy across the simulated landscape, therefore representing the implied indirect solar energy. The model incorporates incoming incident radiation, which varies based on time and geo-location, topographical shading due to local and distant terrain features, and object shadowing due to buildings, vegetative canopies, or any other landscape object casting a shadow.

Penumbra can process landscapes under either stand-alone mode or model-coupled mode. Penumbra running in stand-alone mode provides dynamic solar energy representations across a static landscape. This can be useful to quantify the solar energy state of a landscape for the time period the spatial data represents. Running in coupled mode an ecosystem model could provide Penumbra runtime updates of a changing landscape (e.g. vegetation height). Penumbra in return would provide updates of shade fraction or incident solar energy reaching the ground and water body surfaces. These types of simulations would be especially useful for estimating the future state of a proposed land management plan that is expected to dynamically change the shading elements, such as effects of riparian forest buffer retention or forest plot thinning versus clear-cutting.

Penumbra output may be especially useful to improve other ecosystem model simulation accuracy or to enhance understanding for decision makers regarding vegetation management, stream management or overall landscape health. Penumbra is designed to quantify radiative variability across diverse landscapes through time to help scientists and stakeholders understand the potential consequences of land management on human populations, aquatic environments, and terrestrial habitats.

## Materials and methods

To achieve a balance between computational tractability and the capture of landscape spatial complexity, Penumbra modeling tactic blended two spatial methods: 1) the ray tracing approach with, 2) a two-dimensional cell-to-cell marching tactic. The combined methods retain the three-dimensional approach found in ray tracing, yet within an explicit two-dimensional grid possessing an implicit third vertical dimension.

### Model overview

**Fig 1:**
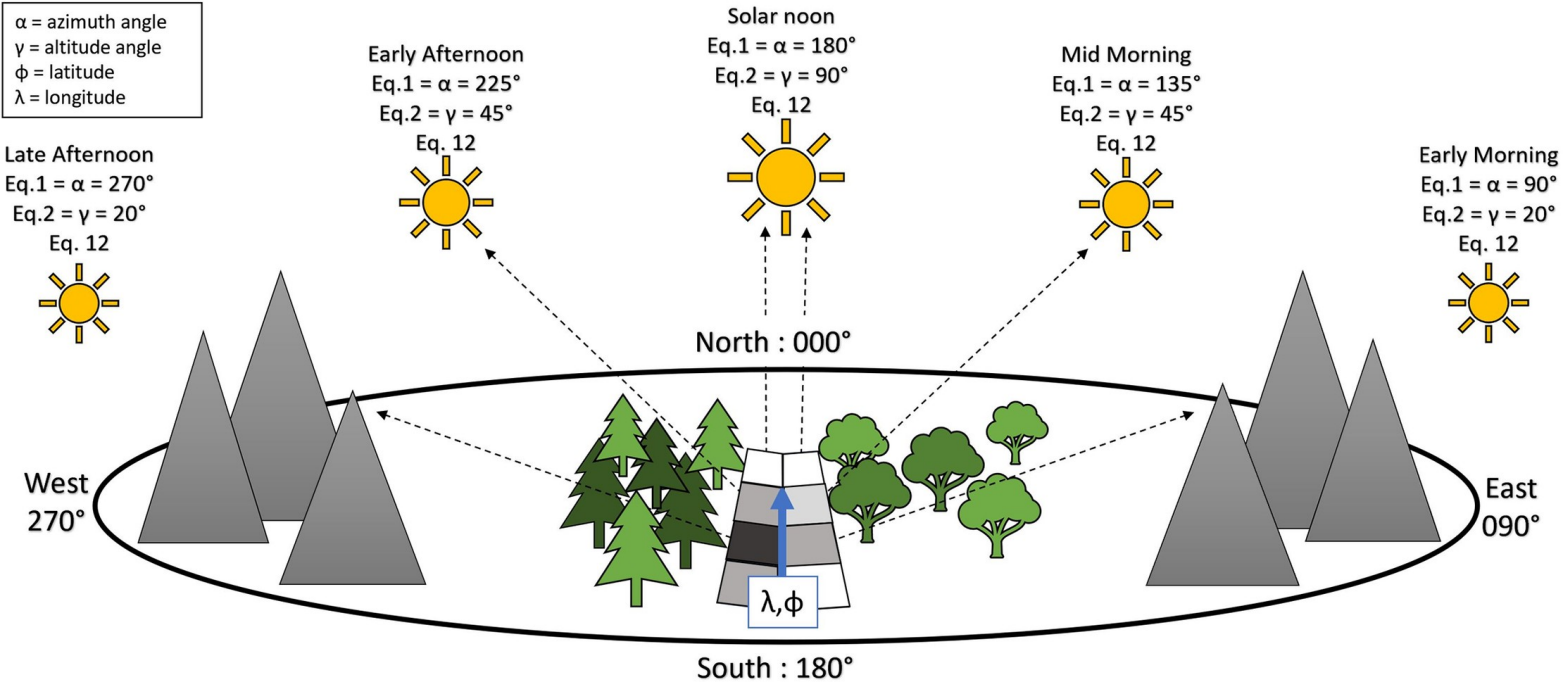
Summary of Penumbra model solar energy tracking. Penumbra's approach to shade modeling for both object and topographic shadowing. A representative set of solar angles are presented, where the cell shade level corresponds to the arrow associated with that cell. Landscape and objects the arrow intercepts dictates the shadow effect upon the cell. Equation numbers correspond to equations described under the Processing Steps.

Penumbra is a spatially distributed, gridded model capable of processing landscapes based on user-defined spatial inputs and temporal parameters. Penumbra assesses individual cells for both object and terrain shadowing that, within a continuous grid coverage, would collectively cover the entire landscape (Fig 1).

**Fig 2:**
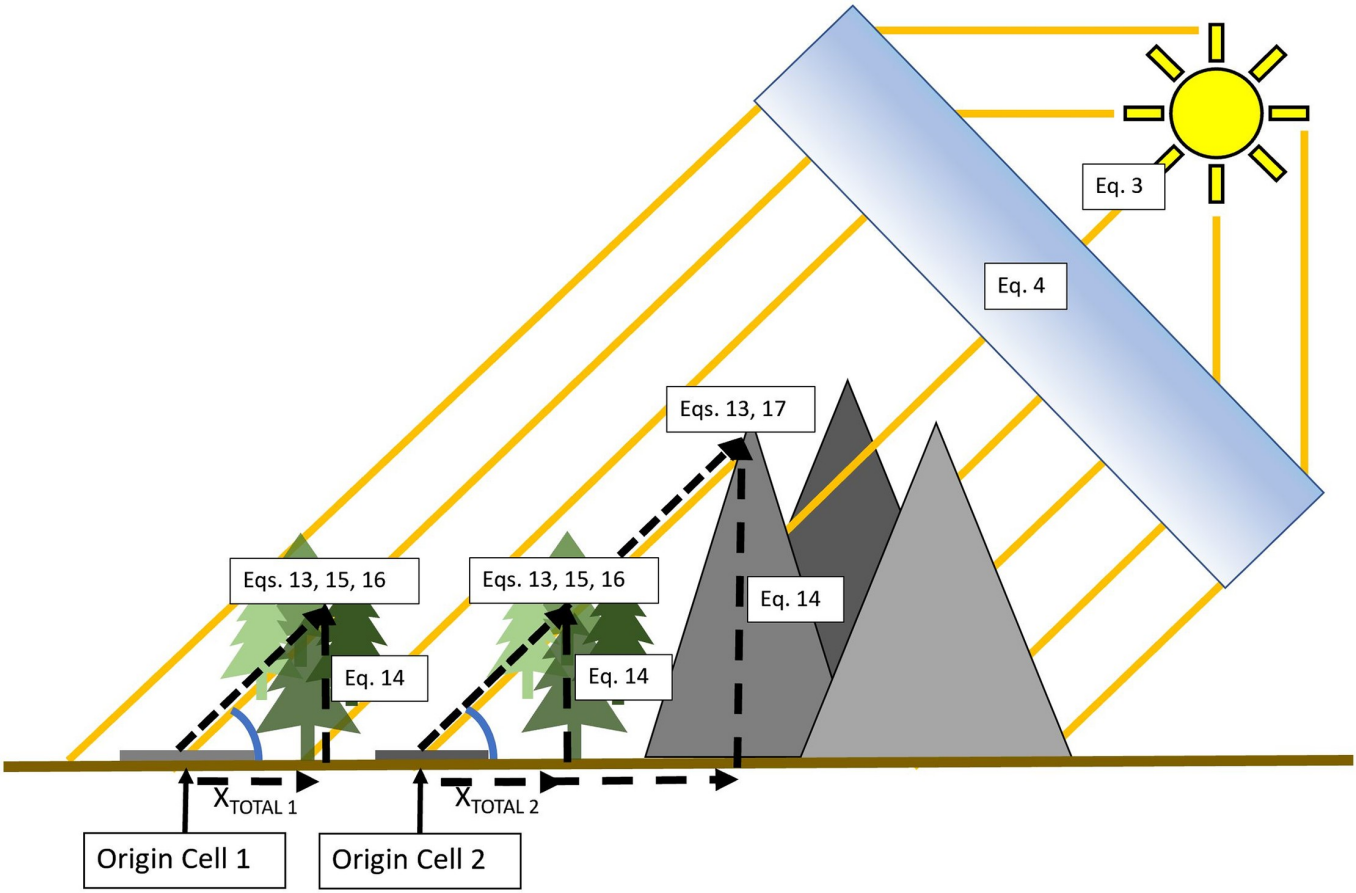
Spatially tracking of solar ray for the *x* and *y*, and *x* and *z* dimensions. Penumbra's solar ray approach to shade modeling for both object and topographic shadowing. Equation numbers correspond to equations described under Processing Steps.

Penumbra simulates the spatial arrangement of ground-level incident solar energy through three independent phases: clear-sky solar energy reaching the landscape, landscape object shadowing, and landscape topographic shadowing. These calculations are performed independently for each cell in a gridded landscape. Penumbra assumes that for any given timestep the environmental lighting comes from a single distant source causing spatially uniform light dispersion [10] (Figs 1 and 2) (Eq 12). This assumption allows Penumbra to assess light ray interference as a reduction of the total available solar energy due to spatially distributed topographic shade and landscape object shade.

The cell resolution of a simulation dictates how detailed objects can be represented in the *x* and *y* dimensions. An object’s *z*-dimension vertical height is representant, yet the object’s structure is a voxel (volume element) with uniform light transmittance throughout; therefore is an implicit representation of the landscape object. Penumbra can simulate an object’s vertical *z*-dimension as either a single voxel, or as two stacked voxels with varied transmittance. The stacked voxel option allows the user to represent a tree’s dominant canopy structure along with a more open understory representation as two uniquely different structures.

#### Inputs

Penumbra requires a small set of model parameters and spatial input data (Table 1). The simulations temporal grain and temporal extent are defined using a start Julian day, stop Julian day, and year range. Penumbra functions under two timing schemes: 1) the frequency of solar angle assessments and 2) the regularity of data aggregation per output. The sun’s positional intervals are defined by the temporal grain (Daily-Grain). This Daily-Grain can be set to any span from one minute up to one full day. Penumbra retains spatial shade and irradiance data arrays through internal aggregation per timestep, then exports the aggregated data per aggregation timestep. Shade data is output as a fraction. Solar energy can be output as the average or accumulated energy.

**Table 1:** List of Penumbra inputs, parameters, and outputs.

Required Inputs	Optional Inputs	Outputs
Start/Stop Julian Day and Year	Forced Start/Stop Times	Ground-level Irradiance
Temporal Grain	Daily Transmittance Map	Total Shade %
Temporal Aggregation	Daily % Cloud Coverage	Object Shade %
Digital Elevation Model (DEM) ^a^	Area of Interest Mask	Topographic Shade %
Normalized Digital Surface Model (nDSM) ^a^	Cell Data Writer Position	Cell Data Values
Object Transmittance Model (OTM) ^a^	Pseudo Height Adjustment	

^a^ Optional: simulation of only terrain or only object shading is feasible. If the DEM or nDSM are excluded, any shadowing that would occur is being excluded. DEM and nDSM are required for complete (topographic and object) shading to be simulated.

Topography is represented using digital elevation data (DEM) data. A spatially equivalent normalized digital surface model (nDSM) represents landscape object heights and is coupled with an object transmittance model (OTM) representing each cell’s light transmission reduction factor scaled 0 through 1. This light reduction should correspond to the landscape object each corresponding cell represents.

Currently, spatial input data are provided as ESRI ASCII Grid format [11]. When both a DEM and nDSM are provided, topographic and object shadowing are simulated; when only a DEM or nDSM is provided, only topographic or object shadowing will be simulated, respectively. Conversely, topographic and object percent shade can be concurrently simulated, yet independently exported. This unique model data output flexibility allows Penumbra simulations to isolate the effect of topographic or object shadowing.

The DEM and nDSM can be obtained through several geographic information science (GIS) approaches. Aerial LiDAR data can be utilized to obtain fine resolution DEM and nDSM data, but Penumbra can also simulate lower resolution nDSM data obtained from species biomass or age relationships. When lower resolution nDSM data are used, the corresponding OTM should reflect the upscaled representation of the object. Recently developed methods for estimating the appropriate light reduction due to landscape objects (trees, shrubbery, etc.) allows for estimation of spatially heterogeneous light transmittance due to approaches like the Light Penetration Index, and similarly the Laser Penetration Index [12–13]. All combined, Penumbra simulations track the accumulation of light attenuation through objects and upon the ground surface; thus, at fine scales simulations can be viewed as modeling tree shadowing, yet at coarser scales be viewed as modeling aggregate stand-level shade.

#### Processing steps

Penumbra processes the landscape per timestep by calculating the current solar position and corresponding clear-sky solar energy, estimating landscape shade by traversing the gridded data space through a method called the *walking algorithm* (described below), and aggregating the results. The topographic and object shade fraction data arrays are multiplied against the clear-sky solar energy to calculate spatially distributed ground-level incident solar energy.

#### Solar angles

Solar azimuth and altitude are calculated at time intervals dictated by the simulation input Daily-Grain. Azimuth (α) and altitude (γ) are calculated by:

α=arccos(EST×ΔJDay−λ) (1)

γ=arcsin(cosφ×cosδ×cosΩ)+sinφ×sinδ (2)

where longitude is λ, latitude is φ, declination is δ, hour angle is Ω, Earth sidereal time is EST, and ΔJDay is the current Julian day minus the January 1^st^ Julian day. The azimuth and altitude algorithms were developed by Holbert and Srinivasan and here implemented in Java [14]. Implementation testing results can be found in S1 Fig.

#### Clear Sky Solar Energy

The clear sky solar energy (EI; W/m^2^) is the quantity of solar radiation reaching the Earth’s atmosphere on a given Julian day for a given geo-location. Per timestep the EI is calculated using the landscapes centroid latitude and longitude. EI is calculated using the following equation [15], where S_sun_ is the solar constant and n is the Julian day.

$$EI=Ssun\times 2\pi \times (\frac{n}{265.25})$$ (3)

This algorithm was developed by ITACA; here implemented in Java [15]. Implementation testing results can be found in S2 Fig.

#### Cosine effect and calibration on solar energy

The EI is reduced to account for the solar cosine effect caused by the solar altitude angle using the following equation, where Θ is the solar altitude and EI. Calibration parameter CS_CALI_ (described below) is applied here.

$$EI_{\cos}=\cos\Theta \times EI\times CS_{CALI}$$ (4)

With the solar angle cosine effect and calibration local peak irradiance accounted for, this resulting EI_cos_ is Penumbra’s clear-sky solar energy value. Here irradiance is specifically mentioned due to Penumbra’s default solar energy units being Watts/meter^2^.

#### Walking algorithm

**Fig 3:**
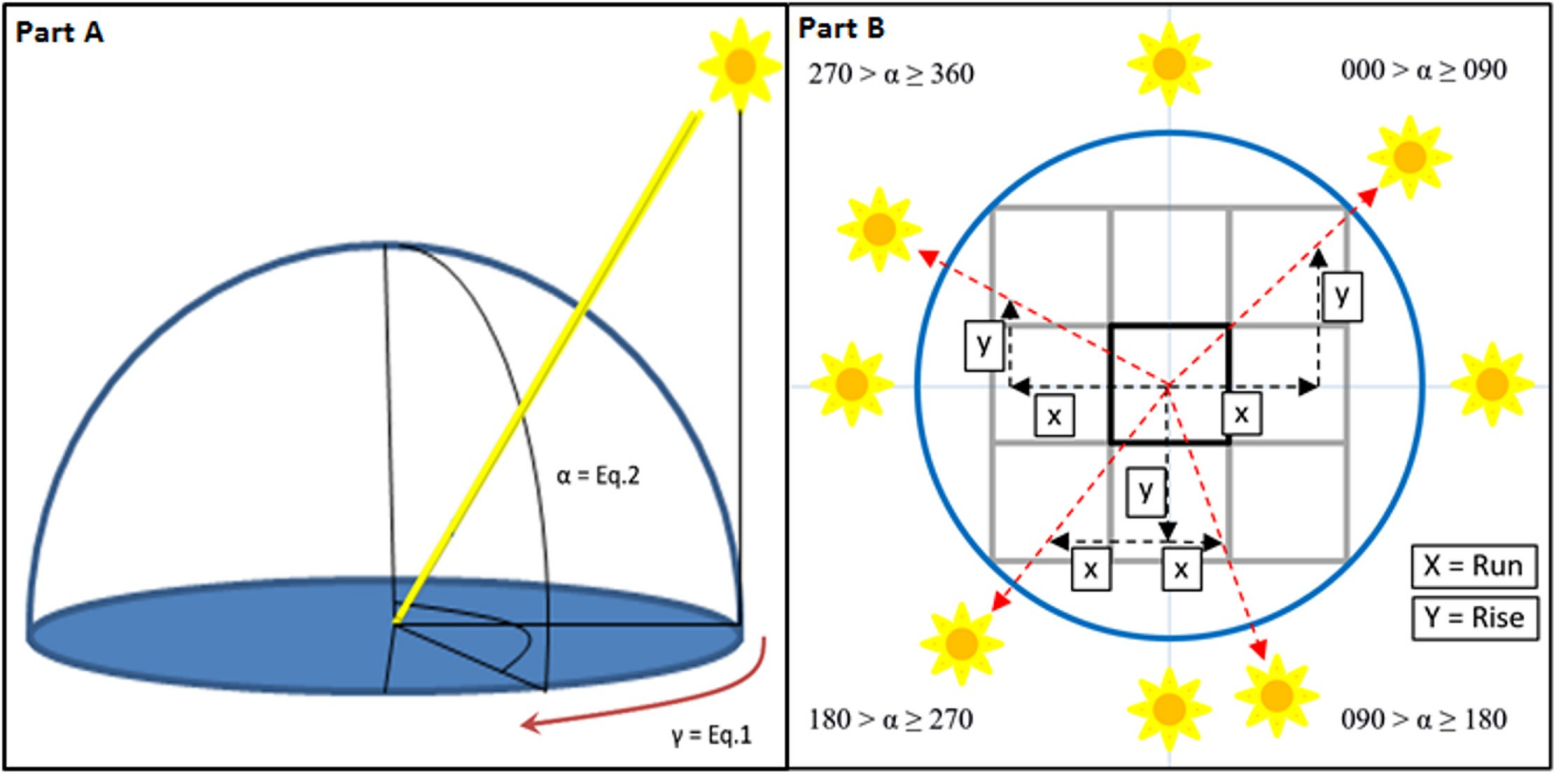
Solar angles and the *walking algorithm*. Solar azimuth (α) and altitude (γ) are calculated to control the direction the *walking algorithm* traverses the landscape. The *walking algorithm* azimuth rise (Y) and run (X) conversions with example sun positions.

The *walking algorithm*, developed from the Marching Squares and Marching Cubes approaches, combined with the backwards ray tracing approach, traces a light ray’s path from each DEM surface cell toward the sun using each timestep’s azimuth and altitude angles [16–17]. The *walking algorithm* computes fractional shade increase due to the interaction with landscape objects or terrain. The *walking algorithm* itself is a set of methods that control the evaluation of shade across the simulation space (Fig 3), with directional assessment and corresponding equations summarized in Table 2. Shade fraction for both object and topographic shade are tracked simultaneously, yet independently. Each cell’s *walking algorithm* termination event occurs when the tracing of the solar ray’s path has encountered either 1) topographic object, 2) reached the edge of the data space, or 3) reached the precalculated ceiling (a value representing the highest combined elevation and object height within the simulation space).

**Table 2:** Azimuth and altitude angles conformed to a Cartesian coordinate system.

	(Conditional Eqs 5–12)
If azimuth (α) is: 0 > α ≥ 90:	If azimuth (α) is: 180 > α ≥ 270:
*Run* = |*sin*(*α*)×*v*| (Eq 5)	*Run* = −1×|*sin*(*α*)×*v*| (Eq 6)
*Rise* = −1×|*cos*(*α*)×*v*| (Eq 7)	*Rise* = |*cos*(*α*)×*v*| (Eq 8)
If azimuth (α) is: 90 > α ≥ 180:	If azimuth (α) is: 270 > α ≥ 360:
*Run* = |*cos*(*α*)×*v*| (Eq 9)	*Run* = −1×|*cos*(*α*)×*v*| (Eq 10)
*Rise* = |*sin*(*α*)×*v*| (Eq 11)	*Rise* = −1×|*sin*(*α*)×*v*| (Eq 12)

The sun’s azimuth and altitude angles are used to derive the rise and run direction that will dictate the *walking algorithm*’s direction away from each cell. The above equation sets function under the four Cartesian angular conditions where α is the timesteps azimuth angle and *v* is the assessment step distance that combined in Eqs 5 through 12 determine the rise and run parameters (Table 2). Variable *v* is distance stepped per *walking algorithm* movement and is defaulted to one quarter the simulation’s cell resolution. The reasoning and benefit of the rise/run/elevation relationship over direct use of the azimuth and altitude is a reduction of repeated trigonometric calculations, which if repeatedly performed are computationally expensive.

#### Solar ray height

As the *walking algorithm* traverses the landscape, each encountered cell is assessed for both topographic and object shadowing upon the origin cell. The sun’s altitude determines the solar ray vertical rise (Solar_VRC_) based on the distance traveled from the origin cell, where γ is the current solar altitude and X_TOTAL_ is the *walking algorithm’*s current total distance between the origin cell and currently encountered cell.

$$Solar_{VRC}=\left|\tan(\gamma )\right|\times X_{TOTAL}$$ (13)

The Solar Vertical Rise Component (Solar_VRC_) represents the absolute height from the initial cell, while the current Solar-ray Absolute Height (Solar_AH_) represents the relative height between the ray’s current height and the corresponding ground elevation (Fig 2).

$$Solar_{AH}=OriginCellElevation+Solar_{VRC}$$ (14)

#### Object shade reductions

As the *walking algorithm* encounters a new cell, that cell’s landscape object interaction is assessed by subtracting the object’s absolute elevation by the Solar_AH_ to obtain the Object Delta Height (O_DH_); the z-dimensional difference between object height and solar ray (Fig 2).

$$O_{DH}=Solar_{AH}-ObjectHeight$$ (15)

If the O_DH_ is positive, the solar ray passed over the object. If the O_DH_ is negative, the solar ray penetrated through the object. As each landscape object’s O_DH_ is encountered, that object’s associated transmittance value is applied to the accumulated solar transmittance in a multiplicative manner. When the *walking algorithm* is terminated the resulting accumulated solar transmittance value is the current origin cell’s final fraction of illumination for that timestep. Object Light Deduction (O_LD_) is the multiplicative of the all encountered cells Object Transmittance (OT) reductions is assigned to that origin cell (Fig 2).

$$O_{LD}=\prod_{i=0}^{n}OT$$ (16)

The *walking algorithm* carries out the above Eqs 5–7 for every cell in the grid space, then summarizing the assessment using Eq 8. When the assessment of the final cell in the two-dimensional spatial domain is complete the data is aggregated into the output arrays and the next timestep is initialized.

#### Topographic Shade Reductions

Topographic shadowing may occur upon the origin cell. Like object shadowing, as the *walking algorithm* encounters a new cell any potential interaction is assessed by subtracting the elevation from the Solar_AH_ to obtain Terrain Delta Height (TDH), which represents the z-dimensional difference between the terrain and ray height (Fig 2).

$$TDH=Solar_{AH}-elevation$$ (17)

If T_DH_ is positive, the solar ray is above the ground and the *walking algorithm* continues assessing the solar path. If T_DH_ is negative, the solar ray penetrated the ground and the *walking algorithm* is terminated. Unlike object shadowing, the aggregation of topographic shadowing does not occur since direct light cannot attenuate through terrain. Instead the Topographic Light Deduction (T_LD_) is calculated based on an inverse relationship of the distance away from the origin cell (X_TOTAL_). At this moment the ground calibration factor (GR_CALI_) is applied.

$$T_{LD}=\frac{1}{X_{TOTAL}}\times GR_{CALI}$$ (18)

If X_TOTAL_ equals one (meaning only one cell was walked), the TLD is equal to GR_CALI_, meaning maximum topographic shade. Any number of additional cells walked would generate a weakening of topographic shade due to the inverse relationship of X_TOTAL_. The inverse effect accounts for scattering of indirect light. The larger the X_TOTAL_ (greater distances), the less topographic shadowing applied upon the origin cell.

#### Shade assessment narratives

With all equations controlling shadowing explained, the following narratives are two examples of shade the assessment process. Example one explains a situation where only object shadowing occurred, whereas example two explains a situation where object and topographic shadowing occurred. Both examples are based on Penumbra default settings. Due to the models spatially distributed grid approach, a fully simulated landscape would result in a shadowing pattern mimicked in the two by eight grid pattern (Fig 1).

1. For situation one, first the solar position and clear-sky solar irradiance are calculated using Eqs 1–4. The direction the *walking algorithm* takes for grid traversing is determined with Eqs 5–12. Per new cell encountered by the *walking algorithm*, the height of the solar ray and the relative height relationship between the ray and landscape object are calculated using Eqs 5 and 6. That final Solar_AH_ value is used to assess the ray position relative to the landscape object. If determined shadowing is occurring based on the current solar angles, the accumulated shadowing effect is summarized with Eq 8. The *walking algorithm* would be terminated based on the ray reaching the edge of the spatial extent or reaching the precalculated ceil for the landscape.
2. For situation two all calculations apply. The singular difference is the termination event, which would occur when the *walking algorithm* determined the solar ray penetrated the terrain. Solar ray penetrating the ground would be determined with Eq 9 when T_DH_ is found to be negative. The level of shadowing from terrain would be calculated with Eq 10.

#### Graphic processing unit (GPU)

The use of a GPU for computational processing is an option in Penumbra. The GPU module replaces the *walking algorithm*, which is the most processing intensive aspect of Penumbra. The GPU approach is based on a computer graphics process known as shadow mapping (the term “shadow” is coincidental), which simulates obfuscation of a surface or object from a light source by rendering a scene from the light source’s viewpoint and generating a lookup table from the subsequent render results [18]. The lookup table is known as a shadow map, and each point in the scene can evaluate against the map by testing its distance from the light source versus the shortest distance recorded in the map [19].

As previously mentioned, Penumbra assumes that light rays are traveling along parallel paths; therefore, an orthographic projection can be used for the sun’s viewpoint, guaranteeing that each ray into the scene only travels through a single row of pixels. The GPU utilizes “shaders” (GPU term “shaders” and Penumbra meaning of “shade” are homonyms) to both quickly render the shadow map and to perform the shadow lookup for each grid point in a massively parallel fashion [18]. However, there is an implicit overhead to moving data to and from the GPU memory. When processing small datasets, the central processing unit (CPU) bound *walking algorithm* will often exceed the GPU shadow mapping approach in runtime performance. When processing landscapes with many spatial or temporal units, the parallel nature of the GPU allows for the shadow mapping approach to produce superior runtime performance. For landscapes characterized at high resolution or representing large spatial extents, and simulations set to a small temporal grain, the GPU is a viable option to shorten simulation runtime requirements.

#### Outputs of shade and incident solar energy

Penumbra can provide representations of landscape illumination as: object shade, terrain shade, total shade (multiplicative of object and terrain shade), or ground-level incident solar energy. All these forms can be output in isolation or together.

Shade outputs are all fraction based. Penumbra internally tracks the deduction of light as a fraction in the Topographic Light Deduction (T_LD_) and Object Light Deduction (O_LD_) data arrays. Spatial shade can be generated per aggregation output by inverting the O_LD_ and T_LD_ data to create Topographic Shade (T_SHADE_) and Object Shade (O_SHADE_), where 1 represents complete shade and 0 represents no shade.

$$T_{SHADE}=1-T_{LD}$$ (19)

$$O_{SHADE}=1-O_{LD}$$ (20)

Total shade (Shade_TOTAL_) is the multiplicative of T_LD_ and O_LD_.

$$Shade_{TOTAL}=T_{SHADE}\times O_{SHADE}$$ (21)

Energy units default to Watts per meter^2^ (W/m^2^), but can be set to report as micromoles/meter^2^/second (μmoles/m^2^/s). The resulting ground-level energy (Energy_RES_) is the multiplicative outcome of Shade_TOTAL_ (Eq 21) and EI_Cos_ (Eq 12), and is the result of all the spatially distributed shadow reductions and all solar energy reductions (Fig 2).

$$Energy_{RES}=Shade_{TOTAL}\times EI_{COS}$$ (22)

Energy_RES_ relies on EI_Cos_, which incorporates the sun cosine effect, atmospheric reductions, along with Shade_TOTAL_ representing all terrain and object shading. Energy_RES_ ultimately represents the final spatially distributed incident ground-level solar energy.

#### Calibration process

Penumbra calibration parameters exist to improve simulation predictive skill. All calibration parameters are scaled from one to zero, where a value of one applies no change and any lower value retains that level of fractional influence. These parameters are applied to data in a uniform and multiplicative manner. The CS_CALI_ (Clear Sky solar energy calibration factor) is an essential parameter. The CS_CALI_ calibration adjusts the landscapes clear-sky solar energy, which accounts for reductions due to atmospheric conditions that cause light scatter and refraction. The TR_CALI_ (Transmittance calibration factor) uniformly shifts the OTM, while the GR_CALI_ (Ground Effect calibration factor) uniformly shifts the influence of topographic shadowing. The capacity that TR_CALI_ and GR_CALI_ have to improve model results are dependent on the proper representation of the initial OTM values and terrain shade reduction values.

Penumbra calibration is a multistep process. The first run is a baseline for comparison, and each subsequent run focuses on one additional calibration parameter. A Penumbra initial run has GR_CALI_ set to 0.5 and all other calibration parameters set to 1.0. Using the initial simulation output, Penumbra’s peak solar energy is calibrated to the observed peak solar energy using CS_CALI_ (Eq 15). The CS_CALI_ calibration adjusts the difference between the observed peak solar energy (PSE_OBS_) and the simulated peak solar energy (PSE_SIM_). The PSE_OBS_ value is obtained from the peak irradiance value within the observed dataset utilized by the model user. CS_CALI_ helps account for regional atmospheric peak clear-sky solar energy deduction caused by air particulate.

$$CS_{CALI}=1-\left(\frac{\left(PSE_{SIM}-PSE_{OBS}\right)}{PSE_{OBS}}\right)$$ (23)

Subsequent Penumbra runs address the impact of shadowing caused by landscape objects and terrain. Similar to the adjustment of the PSE_OBS_ to fit to the PSE_SIM_, the TR_CALI_ and GR_CALI_ calibration parameters are adjusted to improve shadowing and solar energy assessments. Unlike Eq 15, which only matches observed to simulated peak solar energy values, the TR_CALI_ and GR_CALI_ parameters help improve the overall representation of spatial light attenuation for all time steps. Therefore, the model user is relying on predictive skill statistics and plotted visual fit of the observed versus simulated data to adjust TR_CALI_ or GR_CALI_.

Topographic and object shading can be collective in nature, causing an interactive influence within the observed data. This interaction can make sequential calibration difficult due to a TR_CALI_ or GR_CALI_ shift impacting the opposing topographic or object shades influence on Shade_TOTAL_ (Eq 13). General site knowledge can help guide the adjustment of these two calibrations parameters. The dominant influence should be calibrated first.

## Results

Penumbra was tested against observed data to guide model calibration and assess model performance. As previously mentioned, the solar angles were tested using U.S. Navy Observatory data and the clear-sky solar energy results were tested using the NSRDB data (S1 and S2 Figs). The O’CCMoN dataset was utilized for Penumbra testing and demonstration of predictive skill due to these data containing periodically monitored solar energy in both open and forested ecological sites [6].

### Model testing sites

The O’CCMoN monitoring project provides continuous Photosynthetically Active Radiation (PAR) observations at hourly increments for eight pairs of forest and open-sites extending along a 200-km transect from the Pacific Coast to over the crest of the Oregon Cascade Range with the last site in the area of Bend, Oregon. Forest-sites are mature stands with well-established canopies. Open-sites are former mature forest stands that had been clear-cut harvested shortly before installation of climate station sensors. The open-site with paired forest-site monitoring allows the direct assessment of two dramatically different environment types that both reside within the same micro-climate.

High-resolution representation of landscape objects at these sites was required to test Penumbra’s ability to model object shadowing from individual trees. Airborne LiDAR data provided these representations. Of the eight potential O’CCMoN paired sites, only the Moose Mountain and Falls Creek O’CCMoN locations had existing LiDAR coverage [20].

#### Moose mountain

**Fig 4:**
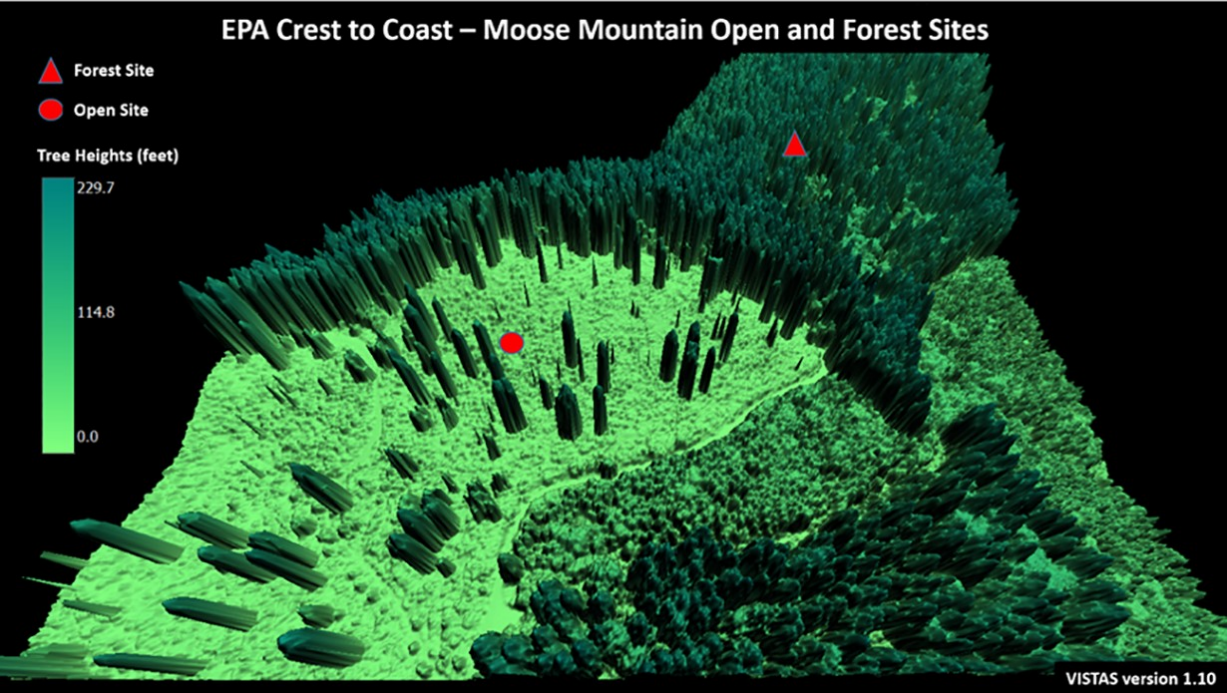
Moose Mountain open-site and forest-site. Sites are part of the Oregon Crest-to-Coast Environmental Monitoring transect (O’CCMoN) dataset. Figure is the gridded 3D representation of the site generated from LiDAR data.

The Moose Mountain Forest-site is a predominantly Douglas-fir forest located in the western Oregon Cascade Range at an elevation of 658 meters above sea level on a southeasterly facing slope. The Moose Mountain open-site is in a clear-cut 460 meters to the southwest at an elevation of 668 meters (Fig 4). PAR is monitored with a LI-COR LI-190SL instrument with the forest-site sensor height being 297 centimeters (cm) and the open-site sensor height being 282 cm from ground level [6].

#### Falls creek

**Fig 5:**
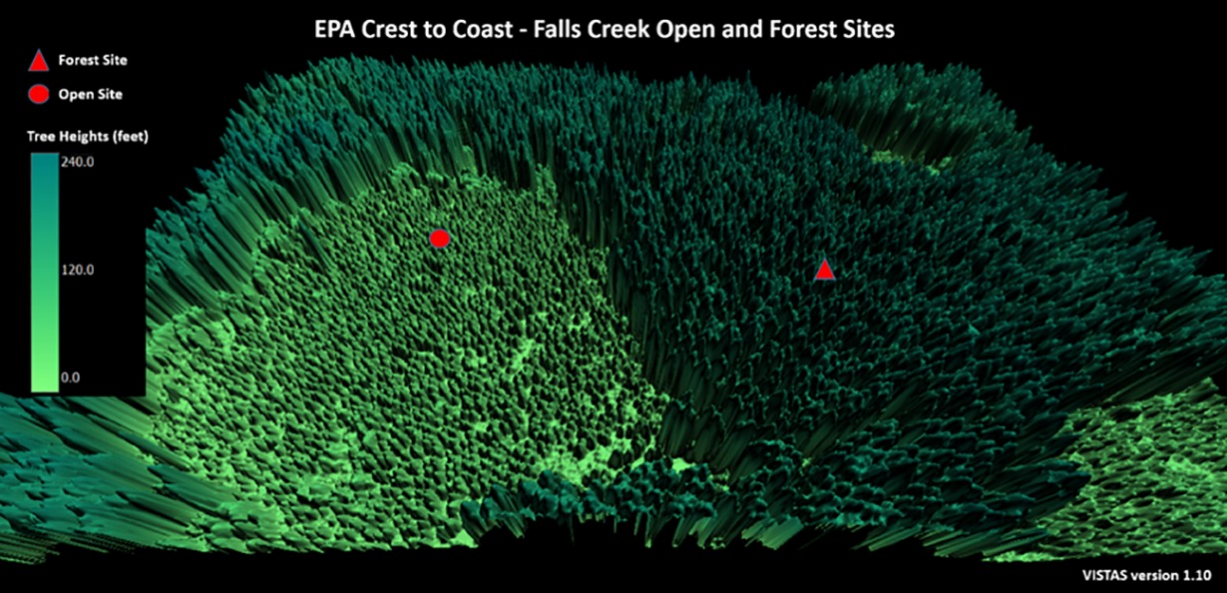
Falls Creek open-site and forest-site. Sites are part of the Oregon Crest-to-Coast Environmental Monitoring transect (O’CCMoN) dataset. Figure is the gridded 3D representation of the site generated from LiDAR data.

The Falls Creek Forest-site is a predominantly Douglas-fir forest. The site location is at an elevation of 528 meters on a slightly northern slope (Fig 5). The Falls Creek open-site is in a recovering clear-cut 328 meters to the west at an elevation of 534 meters. PAR is monitored with a LI-COR LI-190SL instrument with the forest-site sensor height and the open-site sensor height of 334 cm and 353 cm from ground level, respectively [6].

### Model predictive skill

For both locations (four sites in total) the modeled PAR predictions were evaluated against observed hourly data for July 06^th^ through July 09^th^, 2008. Percent-agreement, mean-error, and root mean square error (RMSE) were used to evaluate Penumbra’s simulated goodness-of-fit to the observed field data. Mentioned before, by default Penumbra’s solar energy units are W/m^2^, though the instrumentation collecting the O’CCMoN solar energy data are in PAR (μmoles/m^2^/s). Without pyranometer data for the sites a detailed full light spectrum data conversion could not be carried out. The generalized unit conversion was utilized due to the lack of full light spectrum data [21].

#### Moose Mountain results

The uncalibrated Moose Mountain simulation yielded moderate four-day open-site agreement with a percent-error of 0.511, mean-error of 286.1 (μmoles/m^2^/s) and RMSE of 506.0 (μmoles/m^2^/s) (Table 3). The uncalibrated predictive skill for the forest-site was worse due to no calibration being applied to the global energy and forest transmittance. The forest-site yielded a four-day agreement with a mean-error of -10.0 (μmoles/m^2^/s), RMSE of 77.8 (μmoles/m^2^/s), percent-agreement of 1.55 (Table 3).

**Table 3:** Moose Mountain parameters, initial results, and calibrated results.

Open-site	Initial Run	Calibrated Run	Calibration Parameters	Initial Settings	Calibrated Settings
Percent-agreement	0.52	1.03	CS_CALI_	1.0	0.94
RMSE	506.0	224.6	TR_CALI_	1.0	0.945
Mean-error	286.1	-17.3	GR_CALI_	0.5	0.2
Percent-agreement	1.55	1.86	Sensor heights		
RMSE	77.8	53.8	Open-site	282.5 cm	
Mean-error	-10.0	-15.6	Forest-site	297.2 cm	

**Fig 6:**
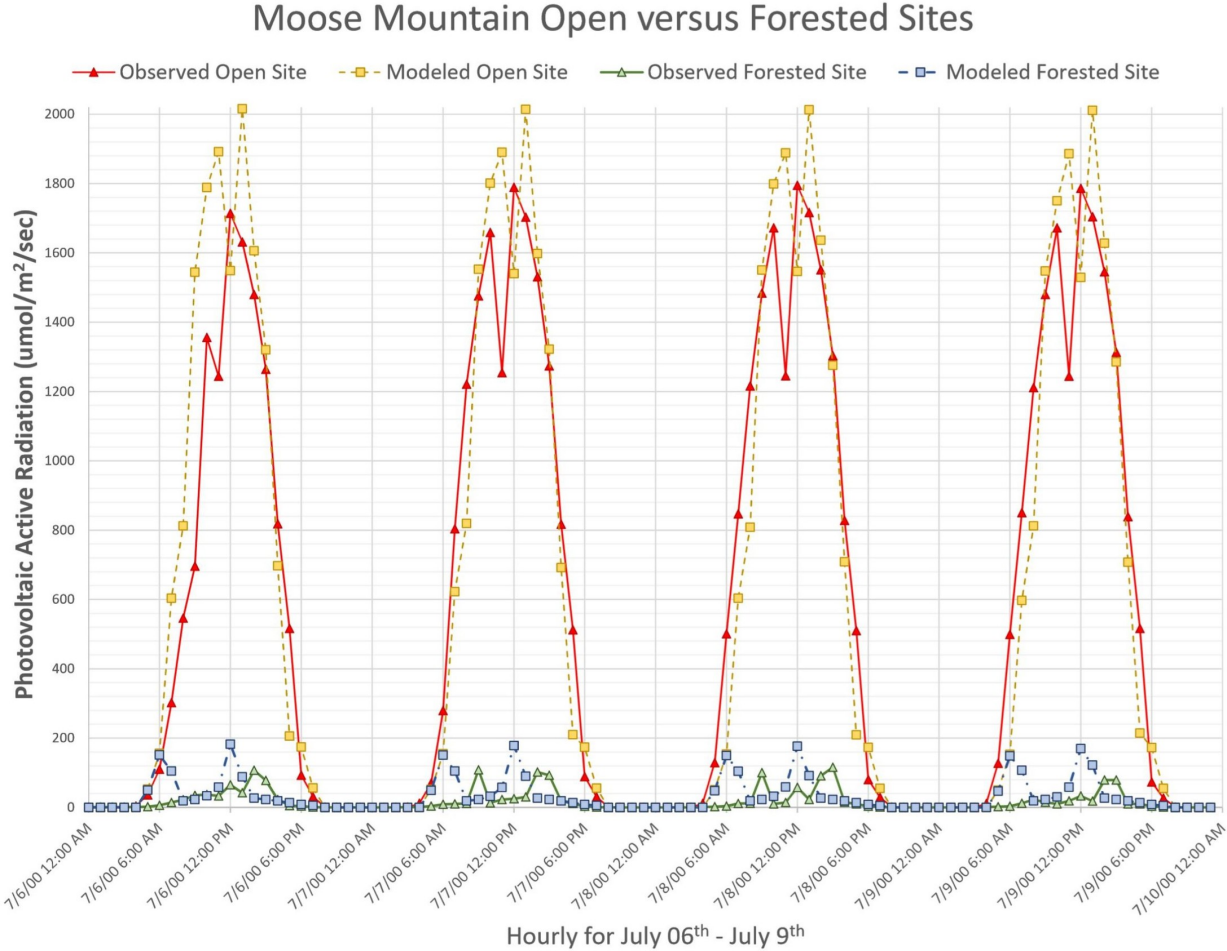
Moose Mountain open-site versus forested-site. These plotted data represent simulated versus observed PAR (μmoles/m^2^/s) for the O’CCMoN Moose Mountain open-site and forested-site. Note the magnitude of solar energy difference between the open versus forested environment.

**Fig 7:**
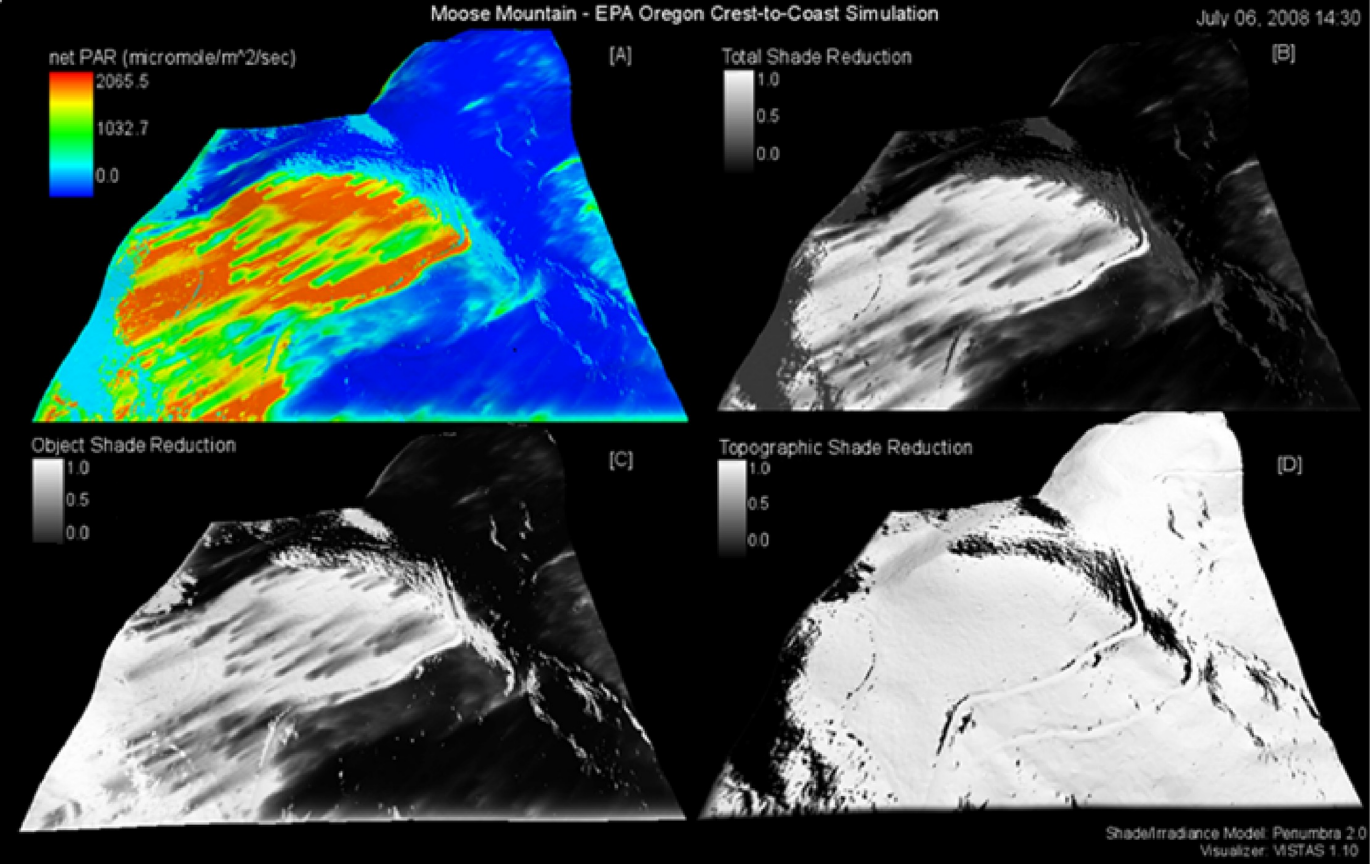
Moose Mountain open and forest-sites simulation still-frame. Still-frame from S1 Video of July 06th, 2008 at 2:30pm. All shade reductions are scaled from 1.0 to 0.0, where 1.0 is no reduction and 0.0 is full reduction. Sub frames: (A) net solar energy as PAR (μmoles/m^2^/s), (B) Total shade reduction (0.0–1.0), (C) Object shade reduction (0.0–1.0), (D) Topographic shade reduction (0.0–1.0). For all (0.0–1.0) shade reductions 1.0 is no reduction and 0.0 is full reduction. See S1 Video for full animation of this simulation.

Calibration of Penumbra greatly improved PAR predictions for the open-site and moderately improved the forest-site (Fig 6); calibration parameters listed in Table 3. The Moose Mountain open-site final calibration provided an excellent observed versus simulated PAR agreement with a RMSE of 224.5 (μmoles/m^2^/s), mean-error of -17.27 (μmoles/m^2^/s), and percent-error of 1.029. The Moose Mountain forest-site final calibration provided a fair percent-error of 1.84, yet a greatly reduced RMSE of 53.78 (μmoles/m^2^/s). The forest-site mean-error had a minor shift to -15.57 (μmoles/m^2^/s) (Table 3). The VISTAS Moose Mountain static frame spatially represents the calibrated run results (Fig 7). *Moose Mountain* video portrays the dynamic simulation (S1 Video). To replicate results, use the provided model and test data (S1 Data).

#### Falls Creek results

The Falls Creek uncalibrated simulation yielded an excellent four-day open-site agreement with a RMSE of 252.6 (μmoles/m^2^/s), mean-error of 59.96 (μmoles/m^2^/s), and percent-agreement of 1.09 (Table 4). The uncalibrated predictive skill for the forest-site resulted with a RMSE of 515.7 (μmoles/m^2^/s), mean-error of -59.90, and percent-agreement of 19.2.

**Table 4:** Falls Creek parameters, initial results, and calibrated results.

Open-site	Initial Run	Calibrated Run	Calibration Parameters	Initial Settings	Calibrated Settings
Percent-agreement	1.09	0.77	CS_CALI_	1.0	1.0
RMSE	252.6	296.8	TR_CALI_	1.0	0.965
Mean-error	-59.96	148.1	GR_CALI_	0.5	0.25
Percent-agreement	19.2	1.51	Sensor heights		
RMSE	515.7	34.2	Open-site	353 cm	
Mean-error	-59.90	-10.4	Forest-site	334 cm	

**Fig 8:**
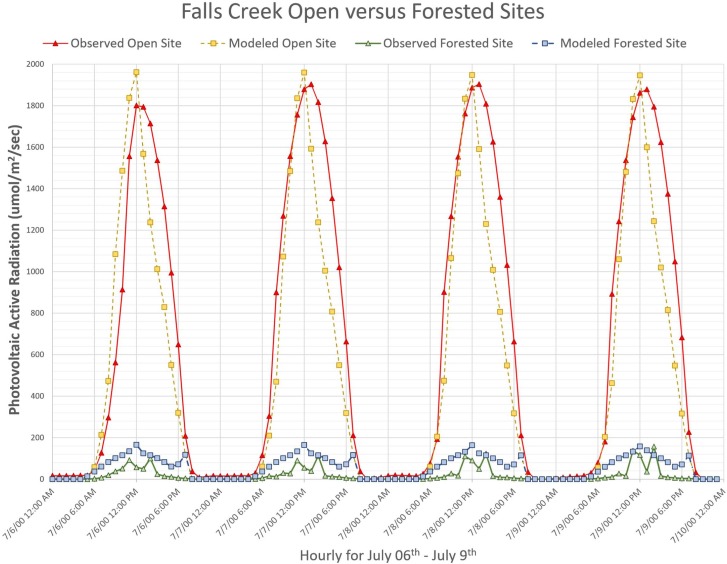
Falls Creek open-site versus forested-site. These plotted data represent simulated versus observed PAR (μmoles/m^2^/s) for the O’CCMoN Falls Creek open-site and Forested-site. Note the magnitude of solar energy difference between the open versus forested environment.

**Fig 9:**
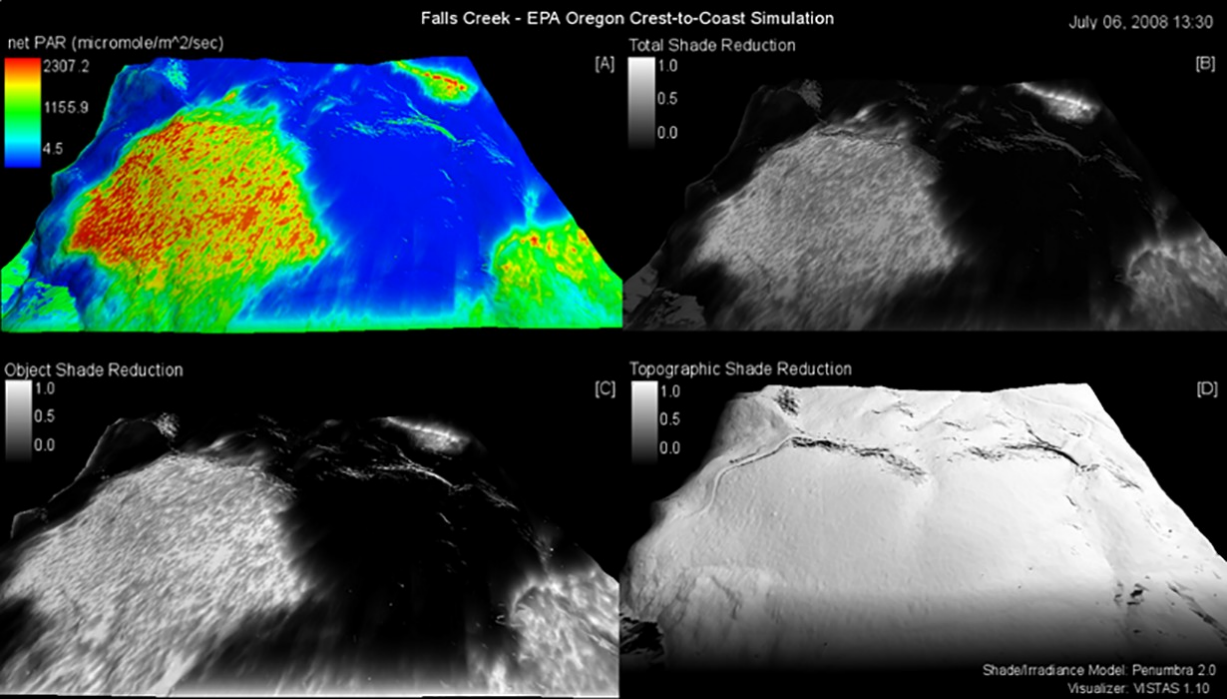
Falls Creek open and forest-sites simulation still-frame. Still-frame from S2 Video of July 06th, 2008 at 2:30pm. All shade reductions are scaled from 1.0 to 0.0, where 1.0 is no reduction and 0.0 is full reduction. Sub frames: (A) net solar energy as PAR (μmoles/m^2^/s), (B) Total shade reduction (0.0–1.0), (C) Object shade reduction (0.0–1.0), (D) Topographic shade reduction (0.0–1.0). For all (0.0–1.0) shade reductions 1.0 is no reduction and 0.0 is full reduction. See S2 Video for full animation of this simulation.

The Falls Creek open-site final calibration yielded a good observed versus simulated PAR agreement with a RMSE of 296.8 (μmoles/m^2^/s), mean-error of 148.1 (μmoles/m^2^/s), and percent-agreement of 0.77 (Fig 8); calibration parameters listed in Table 4. Due to mid-afternoon PAR reductions, the initial run agreement was higher than the final calibration run agreement. The reduced agreement was the result of calibration compromises made to improve the forest-site performance. The VISTAS Falls Creek static frame spatially represents the calibrated run results (Fig 9). *Moose Mountain* video portrays the dynamic simulation (S2 Video). To replicate results, use the provided model and test data (S1 Data).

## Discussion

Penumbra can simulate landscape shade and incident solar energy using standard inputs commonly used in most landscape-scale ecological models or GIS based assessments. Penumbra is one of several models built to simulate environmental solar energy, though Penumbra stands out due to the models approach to simulate complex ground-level solar energy in a simplified form, while not fully compromising complexity of the landscape casting the shade. We acknowledge true objects like trees are more complex than Penumbra is representing. Trees have complex structures that allow light dappling through breaks in the tree crown and tree tops. At large landscape scales the current spatial data dilemma is only airborne LiDAR data is detailed enough to represent tree canopy structure at moderate landscape extents. Even with accurate tree height, LiDAR does not fully represent tree understory structure. Penumbra provides a landscape perspective and modeling approach that fills the niche between the simplistic to complex solar energy modeling approaches, while still providing sufficient detail to capture important spatial and temporal effects on ecological processes.

Spatially distributed landscape scale representations of solar energy allow for the development and improvement of ecological processes that are sensitive to variations in incident solar energy. Solar energy directly drives air temperature and provides energy for leaf photosynthesis, both important drivers of evapotranspiration rates. Solar energy directly warms surface soil and surrounding air temperature. That surface energy transfers down into subsurface soils. The quantity of solar energy available to warm subsurface soils is dependent on the landscape coverage (topography and vegetation) that block direct solar radiation. During rain events the direct heat exchange between substrate and water within a soil column, and heat exchange between surface water runoff and the surface substrate both occur. Vertical and horizontal groundwater energy transfers may indirectly impact stream water temperature, in addition to direct solar radiation impacting stream temperatures. By quantitatively describing the relationship between canopy openness, incident ground-level solar energy and soil temperature, the Penumbra simulation results presented here point to an approach for dynamically simulating effects of upslope disturbances on groundwater and stream temperatures. This achievement could occur by linking Penumbra with an ecohydrological model (e.g., Visualizing Ecosystem Land Management Assessments (VELMA), Regional Hydro-Ecologic Simulation System (RHESSys), Distributed Hydrology Soil Vegetation Model (DHSVM)) that simulates plant-soil-water dynamics within hillslope at watershed scales [2, 22–23].

Penumbra’s flexible temporal parameters allow for landscape assessments to be performed within the context of the user’s research question. Penumbra’s ability to simulate sun position at one temporal grain yet aggregate those timesteps to a greater temporal grain allows for flexible model integration with nearly any other spatiotemporal ecological model. This flexible aggregation allows for data sharing within many research projects involving riparian shading, forest disturbances, photosynthesis, snow melt rates or any other biological process where solar energy has an influence.

Penumbra is subject to the same limitations as most process-based models [2–24]. Penumbra can have lengthy computational runtime requirements, though at typical watershed resolutions and extents Penumbra performs reasonably well. While GPU processing enhancement and direct external model integration assist in reducing the runtime burden, further improvements are needed to do large extent assessments at high-resolution (e.g. 1-meter). For some users, the Java platform in which Penumbra was developed may be a drawback. However, the Java language was selected due to the “write once, run anywhere” perspective, and the language works across many operating systems [25].

Being a process-based model, Penumbra’s testing and uncertainty characterization is a necessity. For assessing Penumbra’s predictive skill, we discovered only the USEPA O’CCMoN dataset possessed the vetted solar energy monitoring needed to compare an open versus forested environment. Other field solar energy datasets we reviewed focused solely on clear-sky or global solar energy monitoring; not sub-canopy solar energy.

The open-site versus forest-site comparisons were demonstrated to reveal how the final forest-site PAR estimates can be greatly improved with minimal to no loss on open-site PAR estimates. We showed the uncalibrated results to highlight how a simplified solar energy model may perform when object and topographic shading are not assessed. Solar energy models that focus solely on global irradiance can only represent impacts on open environments and the surface of forest canopies. In contrast, this initial uncalibrated Penumbra simulation already reduced forest-site ground-level irradiance by roughly half, seen by comparing the open-site to forest-site results (Figs 6 and 8).

Though many locations will not have the observed data needed to perform a rigorous Penumbra calibration, that would also suggest those areas are lacking the data needed for even a basic understanding of ground-level solar energies impact on that environment. Penumbra was developed to function within any environment that a DEM and nDSM can be developed. At the very least, Penumbra shade maps could provide an enhanced understanding of any location lacking a reasonable understanding of solar energy distribution. This capability can also help improve predictive capabilities of ecological models forced with climate models that provide future projections of temperature, precipitation and cloud cover, but with no incident solar energy information.

Penumbra allows for dynamic integration with other ecological models to achieve representations of changing irradiance due to impacts from land-use through time. This can be especially useful for simulating scenarios of alternative land management practices across different regions globally. One particularly important example is characterizing the radiative effects of existing forests and anthropogenic land-use on stream temperature. Furthermore, Penumbra can be integrated with dynamic vegetation growth models to determine the time required to increase riparian shade by some percentage. Urban models could also utilize Penumbra’s object shading feature to determine the spatial impacts of urbanization on irradiance and heat loads within urban centers

## Conclusions

Our field tests of the Penumbra model demonstrate its capabilities for accurately representing spatially-distributed shade and incident ground-level solar energy. For example, at several experimental study sites in Oregon, mean errors in simulated ground-level irradiance measured at open and closed canopy forest sites ranged from -10.4 to 148.1 (Watts/m^2^), or -XX to XX percent of observed values. The largest relative errors were for closed canopy forests, where solar energy was very low, and the light environment was most heterogenous. These results represent a major advance for conducting landscape-scale simulations of incident solar energy and its attenuation across spatially and temporally complex environments within a reasonable simulation timeline.

By simultaneously accounting for topographic and object shading, Penumbra represents a novel ground-level incident solar energy model that fills an important ecological modeling gap. In so doing, Penumbra provides new opportunities. Scientifically, Penumbra can significantly enhance the development and application of ecological models that have lacked an efficient method for quantifying how spatial and temporal variability in solar energy affects fundamental ecological processes, such as photosynthesis, soil temperature and moisture, snow melt and stream temperature. For environmental planning, Penumbra’s reasonable data requirements, speed and ease of use can facilitate environmental decision making by land managers, policy makers and others seeking to mitigate and/or adapt to the effects of changes in land use and climate. Ongoing work with Penumbra is exploring these opportunities.

## Supporting information

S1 Fig Penumbra versus US Navy Observatory azimuth and altitude angles. Validation of Penumbra simulated azimuth and altitude angles. Estimations compared against the U.S. Navy Observatory for June 21st, 1990 [26]. Azimuth agreed with an r^2^ of 0.9923. Altitude agreed with an r^2^ of 0.9991.
(TIF)

S2 Fig Penumbra versus monitored solar irradiance. Validation of Penumbra simulated extraterrestrial irradiance. Estimations compared against monitored DOE-NSRDB data for Salem, Oregon, USA (44.915960°N, -123.001439°W) [27]. Data represents every hour of the year 1990. Simulated irradiance (Watts/m^2^) agreed with an r^2^ of 0.9577.
(TIF)

S1 Video Moose Mountain. *Moose Mountain*.*wmv* O’CCMoN Moose Mountain site simulation for July 06^th^, 2008 through July 09^th^, 2008. [6, 20].
(WMV)

S2 Video Falls Creek. *Falls Creek*.*wmv* O’CCMoN Falls Creek site simulation for July 06^th^, 2008 through July 09^th^, 2008. [6, 20].
(WMV)

S1 Data Penumbra model with test data. *S1_ModelWithTestData*.*zip* Penumbra model provided as a command line runnable Java jar file, test data for both O’CCMoN locations (Moose Mountain and Falls Creek) with prepared properties files for the open site and forest site, and a README file that provides instructions for running the test data through Penumbra.
(ZIP)
