# Supplementary figures and images for: Penumbra: A spatially distributed, mechanistic model for simulating ground-level incident solar energy across heterogeneous landscapes
Source: PLoS One. 2018 Dec 19;13(12):e0206439. doi: 10.1371/journal.pone.0206439 (PMC6300277; doi:10.1371/journal.pone.0206439)

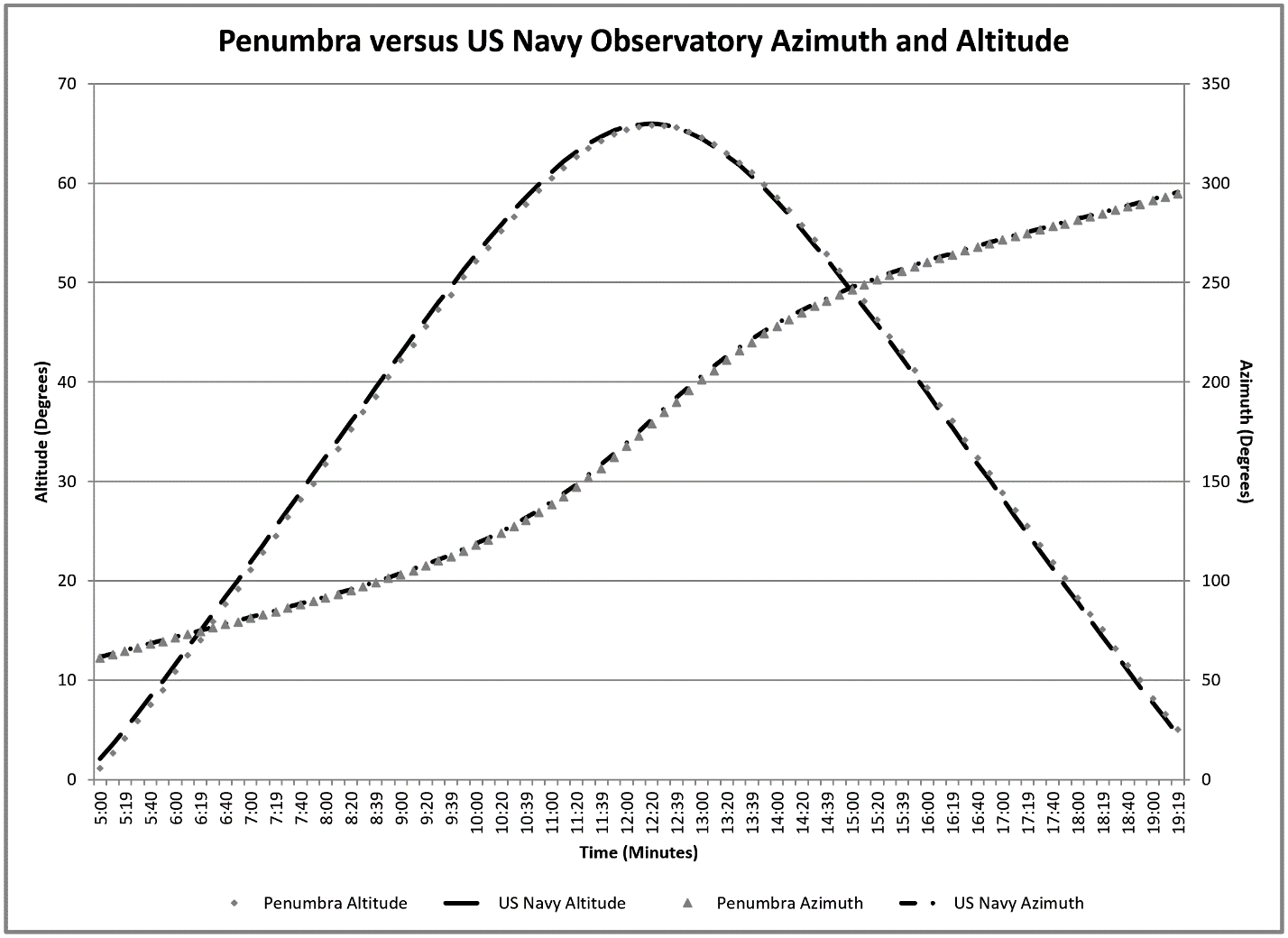

Supplement: S1 Fig — Validation of Penumbra simulated azimuth and altitude angles. Estimations compared against the U.S. Navy Observatory for June 21st, 1990 [26]. Azimuth agreed with an r2 of 0.9923. Altitude agreed with an r2 of 0.9991. (TIF) [file pone.0206439.s001.tif]

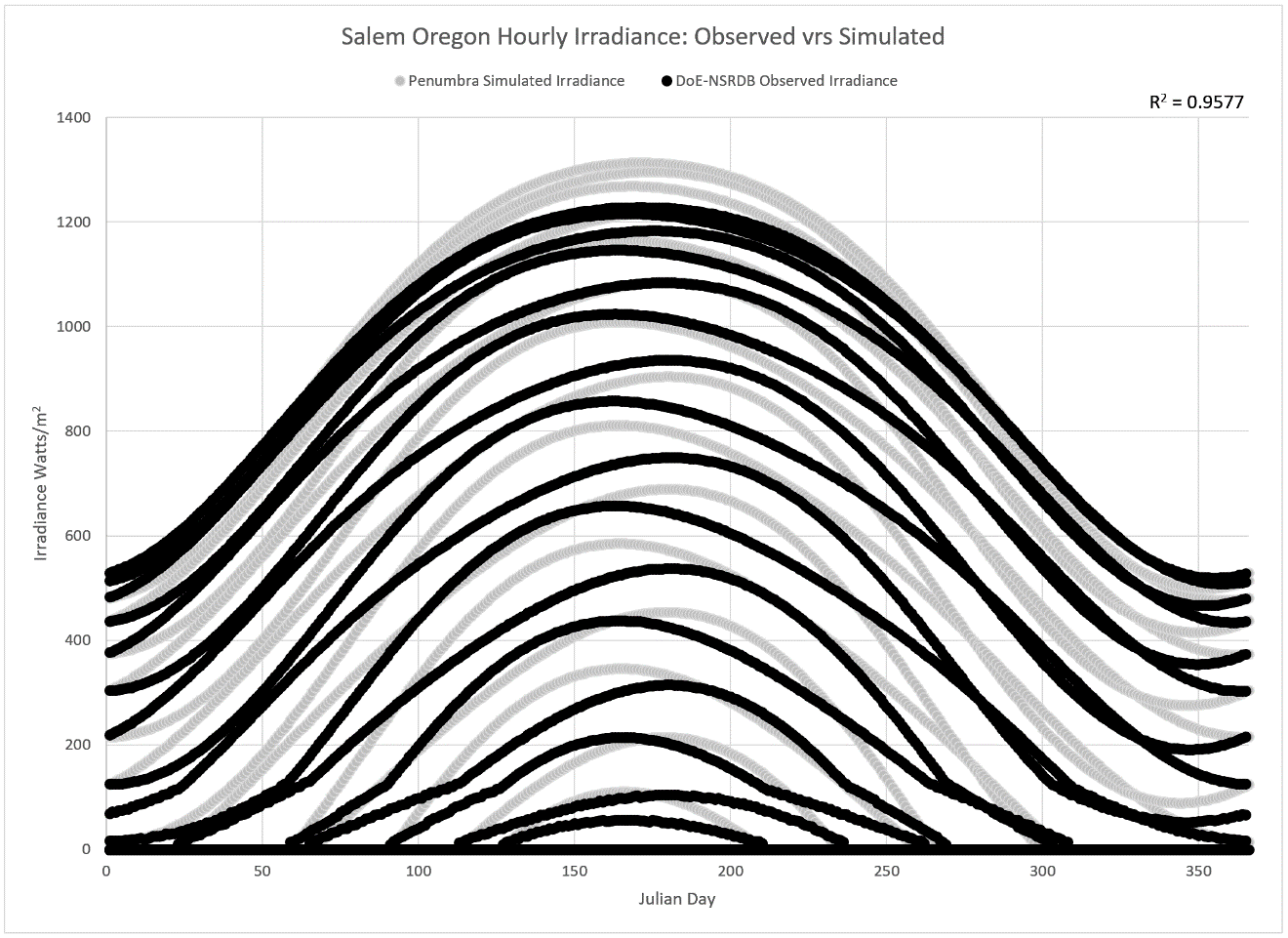

Supplement: S2 Fig — Validation of Penumbra simulated extraterrestrial irradiance. Estimations compared against monitored DOE-NSRDB data for Salem, Oregon, USA (44.915960°N, -123.001439°W) [27]. Data represents every hour of the year 1990. Simulated irradiance (Watts/m2) agreed with an r2 of 0.9577. (TIF) [file pone.0206439.s002.tif]
