# Supplementary material for: Penumbra: A spatially distributed, mechanistic model for simulating ground-level incident solar energy across heterogeneous landscapes
Source: PLoS One. 2018 Dec 19;13(12):e0206439. doi: 10.1371/journal.pone.0206439 (PMC6300277; doi:10.1371/journal.pone.0206439)
Supplement: S1 Data — S1_ModelWithTestData.zip Penumbra model provided as a command line runnable Java jar file, test data for both O’CCMoN locations (Moose Mountain and Falls Creek) with prepared properties files for the open site and forest site, and a README file that provides instructions for running the test data through Penumbra. (ZIP) [file pone.0206439.s005.zip › PenumbraTestData/README_Launching Penumbra with Test Data.pdf]

How to Launch Penumbra and Run the Test Data Used in the Manuscript:

Penumbra: A spatially distributed, mechanistic model for simulating ground-level incident solar energy across heterogeneous landscapes

Provided Files

- Penumbra_ Runnable.jar
- MooseMountain_Forest_Site.properties
- MooseMountain_ Open_Site.properties
- FallsCreek_Forest_Site.properties
- FallsCreek_ Open_Site.properties
- This README file: README_Launching Penumbra with Test Data.pdf
- Summary Results provided in Excel files found in folder "SolarEnergyResults" for all sites.

System Requirements

The Penumbra model was developed in Java. To run the Penumbra.jar, the computer system being used must have Java 1.8 (64-bit) or better. The standard Java JVM memory allocation is enough for these test data. The following writeup utilizes Windows PowerShell. Command line calls from other command prompts would work as well, though syntax may vary.

Fix Directory Structure

Foe each properties file ran, open the properties file and change the "mainDirectory" and "mainOutputDirectory" variables following component "C:\\Users\\Jonat\\Desktop\\" to the fully qualified file path of the location the zipped data was extracted.

Steps to Run Penumbra with Test Data

1. Launch PowerShell
2. Type the following: java -jar (one space between "java" and "-jar")
3. Drag-n-drop the Penumbra_Runnable.jar file in the PowerShell prompt
4. Add a single space by clicking the spacebar
5. Drag-n-drop the MooseMountain.properties file or FallsCreek.properties file in the PowerShell prompt (only one properties file per run)
6. Click the Enter key

Note: If not using PowerShell, the fully qualified file paths to the jar file and properties file must be typed out by hand. Using PowerShell Drag-n-drop ability, PowerShell inserts the fully qualified file paths for the user.

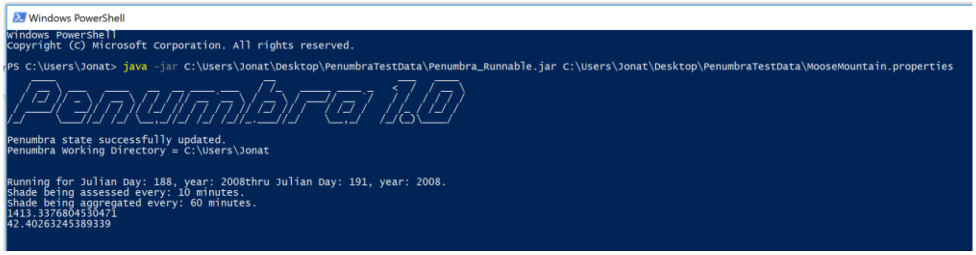

A successful Penumbra launch will include "Penumbra 1.0" and statement "Penumbra state successfully updated.". When backcasting data, Penumbra's aggregation time should match the timing of data observation and collection, as well as the units of the data.
